# Interval Prediction of Remaining Useful Life Based on Uncertainty Quantification with Bayesian Convolutional Neural Networks Featuring Dual-Output Units

**DOI:** 10.3390/s26092592

**Published:** 2026-04-22

**Authors:** Zhendong Qu, Jialong He, Yan Liu, Song Mao, Xiaowu Han

**Affiliations:** 1 Key Laboratory of CNC Equipment Reliability, Ministry of Education, Jilin University, Changchun 130022, China; quzd22@jlu.edu.cn (Z.Q.); hejl@jlu.edu.cn (J.H.); maosong23@jlu.edu.cn (S.M.); 18634874024@163.com (X.H.); 2 School of Mechanical and Aerospace Engineering, Jilin University, Changchun 130022, China

**Keywords:** remaining useful life prediction, uncertainty quantification, Bayesian deep learning, convolutional neural network, interval prediction

## Abstract

RUL prediction methods do not fully account for the uncertainties caused by data scarcity and inherent noise, and they also suffer from low reliability of RUL point estimates. To tackle these challenges, this paper proposes a Bayesian convolutional neural network with dual-output units for RUL interval predictions. The network employs the negative log-likelihood as the loss function. Thanks to its dual-output structure, it not only provides point estimates, but also quantifies the aleatoric uncertainty inherent in the data. During the training process, the CNN is reformulated using Bayesian principles, and the Bayes-by-backprop method is applied to train the network. This transformation converts model parameters from fixed values into random variables. As a result, epistemic uncertainty caused by model inaccuracies and limited data can be quantified. Experimental validation on the IEEE PHM Challenge 2012 dataset demonstrated that the proposed method achieved a higher prediction accuracy than state-of-the-art uncertainty-aware prediction approaches, demonstrating a better applicability in engineering practice.

## 1. Introduction

Prognostics and health management (PHM) is a decision-support system designed to ensure equipment safety and reduce maintenance costs by leveraging physical knowledge, operational information, and data from industrial equipment operation and maintenance [1,2,3]. The prediction of the remaining useful life (RUL) is a core component of PHM technology, serving as a prerequisite and foundation for system maintenance, and it represents a key research focus in the field of reliability engineering.

With the rapid development of sensor technology, increasingly integrated and miniaturized sensing devices are being widely deployed in mechanical systems to monitor equipment health conditions. In such a data-rich environment, deep learning techniques have demonstrated strong capabilities for RUL predictions. Various models, including convolutional neural networks (CNNs) [4], long short-term memory networks (LSTMs) [5], gated recurrent units (GRUs) [6], and graph convolutional networks (GCNs) [7], as well as their variants, have achieved a promising performance in bearing RUL prediction tasks.

However, most existing deep learning models focus on point estimation and fail to quantify the predictive uncertainty, which limits their reliability in practice [8]. In practice, data acquisition is often affected by noise and measurement errors, which are referred to as aleatoric uncertainty [9]. Homoscedastic uncertainty assumes a constant variance, whereas heteroscedastic uncertainty allows it to vary across inputs. Meanwhile, the uncertainty in model fitting caused by factors such as insufficient data or inappropriate model selection is referred to as epistemic uncertainty [10]. To quantify the uncertainties in practical engineering applications, Bayesian neural networks are widely employed in the field of remaining useful life predictions [11]. In Bayesian neural networks, all weights are represented by probability distributions of random variables, unlike traditional deep learning networks, where the weights are assigned fixed values [12]. During the training process, Bayesian neural networks quantify uncertainty through probability distributions, yielding results that are more reliable due to the provision of confidence intervals. This characteristic also facilitates a subsequent analysis and decision-making.

In addition to Bayesian approaches, recent studies have explored uncertainty-aware RUL predictions from different perspectives. Evidential deep learning [13] has been introduced to model predictive uncertainty through evidence theory without relying on sampling-based inference, while deep equilibrium models [14] have been proposed to enhance model representation and stability in RUL estimation. However, these methods do not explicitly provide a unified probabilistic framework for decomposing the predictive uncertainty into aleatoric and epistemic components.

Currently, the variational inference (VI) method is commonly used to train Bayesian neural network (BNN) models. Variational inference [15] employs an approximate distribution, parameterized by θ, to approximate the posterior distribution of network parameters, and iteratively optimizes this approximate distribution by solving an optimization problem. It is widely applicable and converges quickly. Two common forms of VI are the Monte Carlo (MC) dropout method and the Bayes-by-backprop method. Weiwen Peng et al. [16] integrated the MC dropout method with a bidirectional LSTM neural network and a multi-scale convolutional neural network, using dropout to achieve a Bayesian approximation for uncertainty quantification. Gaoyang Li et al. [10] proposed a novel Bayesian framework that combines the MC dropout method with a dual-output neural network. In this framework, the dual-output structure captures the aleatoric uncertainty, while dropout is used to implement Bayesian approximation in the neural network to capture epistemic uncertainty, thereby completing uncertainty quantification. Among the aforementioned methods, MC dropout is widely adopted by researchers for uncertainty quantification due to its relative ease of implementation. However, the MC dropout method can only capture epistemic uncertainty related to weights by randomly activating or deactivating neurons, while neglecting the aleatoric uncertainty caused by inherent noise in the data [17]. Additionally, the MC dropout method has been criticized for its inability to converge as the amount of data increases, a limitation that persists even in simple linear networks [18]. Kumar Shridhar et al. [19] utilized the Bayes-by-backprop method for uncertainty quantification in a BNN-CNN, employing a loss function composed of the sum of the negative log-likelihood and the Kullback–Leibler divergence. Lei Wang et al. [1] proposed a Bayesian large-kernel attention mechanism network for bearing remaining useful life predictions; it employs the Bayes-by-backprop method for network uncertainty quantification. Bayes-by-backprop implements the iterative optimization of the approximate distribution in the variational inference using the backpropagation algorithm [12]. Similar to the MC dropout method, this approach also overlooks the aleatoric uncertainty arising from inherent noise in the data. Yaguo Lei et al. [20] introduced a dual-output Res-ConvLSTM network, trained using a hybrid loss function that includes the mean squared error, log probability density, and confidence interval width definitions. While this method achieves quantification of the aleatoric uncertainty, it lacks a process for quantifying the epistemic uncertainty resulting from inaccuracies in the model’s fitted distribution.

Based on the above analysis, the existing methods still face two major challenges in RUL predictions:

(1) The aleatoric uncertainty is often oversimplified or assumed to be constant; thus, the methods fail to capture its heteroscedastic nature [21].
(2) The epistemic uncertainty is frequently ignored in dual-output structures, resulting in incomplete uncertainty modeling [22].

Although BNNs have been widely used for uncertainty quantification, most existing approaches either focus primarily on epistemic uncertainty or model aleatoric uncertainty separately without a unified framework. As a result, they cannot simultaneously capture both types of uncertainty in a consistent probabilistic formulation.

To address the aforementioned limitations, this paper employs a convolutional neural network (CNN) as the backbone architecture, reformulates it within a Bayesian framework, and incorporates a dual-output structure. Bayesian inference is performed via the Bayes-by-backprop method for bearing RUL predictions and uncertainty quantification. The main contributions of this work are summarized as follows:

1. A unified probabilistic framework is proposed to jointly model aleatoric and epistemic uncertainties for RUL predictions. The dual-output structure enables the modeling of heteroscedastic aleatoric uncertainty, while the Bayesian formulation captures epistemic uncertainty through parameter distributions.
2. A Bayesian convolutional neural network (BCNN) with a dual-output structure is developed, in which model parameters are treated as random variables and optimized via Bayes-by-backprop, enabling consistent uncertainty quantification and an improved prediction reliability.

Compared with existing Bayesian neural network-based methods, the proposed approach provides a unified and principled framework for jointly modeling aleatoric and epistemic uncertainties, enabling more comprehensive and reliable uncertainty quantification for RUL predictions.

The structure of this paper is organized as follows: Section 2 introduces the theoretical foundations of Bayesian deep learning (BDL) networks and variational inference. Section 3 presents the proposed dual-output Bayesian convolutional neural network (BCNN) framework and elaborates on uncertainty quantification for remaining useful life predictions. Section 4 validates the effectiveness of the proposed method on a public bearing dataset and compares it with other uncertainty quantification approaches. Section 5 concludes the paper.

## 2. Methods and Theories

### 2.1. Bayesian Neural Networks

In a classical regression model, the remaining useful life prediction is often formulated as $y=f^{w}\left(x\right)$, where x and y represent the input and output of the system, respectively, and w denotes the parameters of the regression model. The essence of Bayesian deep learning (BDL) lies in preserving the topological structure of the deep learning network while treating its parameters as random variables, thereby enabling uncertainty quantification through probability distributions. This approach retains the superior feature extraction and analytical capabilities of deep learning models, while leveraging Bayesian inference to update and optimize the probability distributions of the model parameters.

For a set of system run-to-failure training trajectories, the dataset is denoted as $D={\left\{\left(X_{i},Y_{i}\right)\right\}}_{i=0}^{N}$, where N is the total number of training trajectories. For the *i*-th trajectory, $X_{i}={\left\{x_{i,j}\right\}}_{j=0}^{N_{i}}$ and $Y_{i}={\left\{y_{i,j}\right\}}_{j=0}^{N_{i}}$, where $x_{i,j}$ represents the input sample at the *j*-th time step of the *i*-th trajectory, $y_{i,j}$ is the corresponding RUL label, and $N_{i}$ denotes the number of samples in the *i*-th trajectory. The Bayesian deep learning (BDL) model is formulated as $y=f^{w}\left(x\right)$, with a prior distribution $p\left(w\right)$. The likelihood function after model regression is defined as $p\left(y_{i}|\;f^{w}\left(x_{i}\right)\right)$. In bearing remaining useful life prediction tasks, this likelihood is often defined as a Gaussian distribution, i.e., $y_{i}\;\sim \;N\left(f^{w}\left(x_{i}\right),I\right)$, where I represents the standard deviation of the likelihood distribution. For newly observed data $x^{*}$, the predictive probability of the model is expressed as:

(1) $$p\left(y^{*}|\;x^{*},D\right)=\int p\left(y^{*}|\;f^{w}\left(x^{*}\right)\right)p\left(w|\;D\right)dw$$

where $p\left(w|D\right)$ is referred to as the posterior distribution. According to Bayesian theory, the posterior distribution is computed as:

(2) $$p\left(w|\;D\right)=\frac{p\left(w\right)p\left(D|\;w\right)}{p\left(D\right)}=\frac{p\left(w\right)p\left(D|\;w\right)}{\int p\left(D|\;w\right)p\left(w\right)dw}$$

It can be observed that the model predictive probability involves an integration problem, and the posterior distribution is generally intractable to compute directly, especially for complex network structures and high-dimensional data. This intractability poses a significant challenge for probability integration. Variational inference (VI) is a commonly used optimization method in machine learning for approximating intractable distributions [23].

### 2.2. Variational Inference Theory

The implementation of variational inference (VI) generally consists of two steps: First, a family of probability distributions $q_{\phi }\left(w\right)$ parameterized by ϕ is selected as the variational distribution family; second, the optimal variational distribution is found by minimizing the Kullback–Leibler (KL) divergence between $q_{\phi }\left(w\right)$ and the true posterior $p\left(w|\;D\right)$ [14]. The KL divergence is defined as follows:

(3) $$\begin{array}{l} KL\left[q_{\phi }\left(w\right)||\;p\left(w|\;D\right)\right]=\int q_{\phi }\left(w\right)\log\frac{q_{\phi }\left(w\right)}{p\left(w|\;D\right)}dw\\ =\int q_{\phi }\left(w\right)\log\frac{q_{\phi }\left(w\right)p\left(D\right)}{p\left(D|\;w\right)p\left(w\right)}dw\\ =\int q_{\phi }\left(w\right)\log\frac{q_{\phi }\left(w\right)}{p\left(w\right)}dw-\int q_{\phi }\left(w\right)\log p\left(D|\;w\right)dw+\int q_{\phi }\left(w\right)\log p\left(D\right)dw\\ =\int q_{\phi }\left(w\right)\log\frac{q_{\phi }\left(w\right)}{p\left(w\right)}dw-\int q_{\phi }\left(w\right)\log p\left(D|\;w\right)dw+\log p\left(D\right)\\ =KL\left[q_{\phi }\left(w\right)||\;p\left(w\right)\right]-E_{q_{\phi }\left(w\right)}\left[\log p\left(D|\;w\right)\right]+\log p\left(D\right) \end{array}$$

In Formula (3), the posterior distribution is expanded for analysis. As a result, the KL divergence can be decomposed into three components. The first part represents the KL divergence values of the variational distribution family $q_{\phi }\left(w\right)$ and the prior $p\left(w\right)$; the second part is to convert the original integral term into the corresponding expected term, in order to facilitate further simplification and implementation using Monte Carlo (MC) sampling in the future; and the third part represents a constant that is independent of ϕ and can be ignored in minimization (3). If $\phi^{*}$ is used to represent the optimization problem, then after the above transformation, the expression of the optimization problem is as follows:

(4) $$\begin{array}{l} \phi^{*}=\arg\min_{\phi }KL\left[q_{\phi }\left(w\right)||\;p\left(w|\;D\right)\right]\\ =\arg\min_{\phi }\left\{KL\left[q_{\phi }\left(w\right)||\;p\left(w\right)\right]-E_{q_{\phi }\left(w\right)}\left[\log p\left(D|\;w\right)\right]\right\} \end{array}$$

The corresponding objective function takes the form of:

(5) $$F\left(D,\phi \right)=KL\left[q_{\phi }\left(w\right)||\;p\left(w\right)\right]-E_{q_{\phi }\left(w\right)}\left[\log p\left(D|\;w\right)\right]$$

Most of the existing references are written in ELBO form, and its expression is as follows:

(6) $$\begin{array}{l} ELBO=E_{q_{\phi }\left(w\right)}\left[\log p\left(D|\;w\right)\right]-KL\left[q_{\phi }\left(w\right)||\;p\left(w\right)\right]\\ =E_{q_{\phi }\left(w\right)}\left[\log p\left(D|\;w\right)\right]-E_{q_{\phi }\left(w\right)}\left[\log q_{\phi }\left(w\right)\right]+E_{q_{\phi }\left(w\right)}\left[\log p\left(w\right)\right] \end{array}$$

In Formula (6), the minimization optimization problem is transformed into the maximization ELBO problem. The $KL\left[q_{\phi }\left(w\right)||\;p\left(w\right)\right]$ term is called the complexity cost, which represents the physical distance between the variational distribution family $q_{\phi }\left(w\right)$ and the model prior $p\left(w\right)$, and it is used to constrain $q_{\phi }\left(w\right)$ to approximate the prior $p\left(w\right)$. The $E_{q_{\phi }\left(w\right)}\left[\log p\left(D|\;w\right)\right]$ term is called the likelihood cost, which constrains the model to fit the training data. In the previous definition, let the prior $p\left(w\right)$ follow a Gaussian distribution with a mean of 0 and a variance of $\sigma_{p}^{2}$; the variational distribution $q_{\phi }\left(w\right)$ is also assumed to follow the Gaussian distribution $w\;\sim \;N\left(\mu ,\sigma^{2}\right)$, where μ and $\sigma^{2}$ represent the mean and variance of the Gaussian distribution, respectively. In the second equation of Formula (6), the $KL\left[q_{\phi }\left(w\right)||\;p\left(w\right)\right]$ term is written as the logarithmic expected form of the variational distribution $q_{\phi }\left(w\right)$ and the prior $p\left(w\right)$, respectively. The purpose of this is to estimate the expected value through MC sampling.

### 2.3. Parameter Resampling

The MC sampling method estimates the expected value through multiple sampling processes, and the corresponding expression can be written as:

(7) $$Loss=-\left\{\sum_{s=1}^{N_{train}^{s}}\left[\log p\left(D|w^{\left(s\right)}\right)-\log q_{\phi }\left(w^{\left(s\right)}\right)+\log p\left(w^{\left(s\right)}\right)\right]\right\}$$

In the process of sampling w, we need to sample w from $N\left(\mu ,\sigma^{2}\right)$. If we directly sample w, because the sampling action is discrete, the result is not differentiable. Therefore, in reality, there is no gradient information when used for backpropagation, and it is impossible to update the parameter gradient. To solve such problems, reparameterization techniques are often used to transform the sampling process into obtaining ε from the standard normal distribution $N\left(0,1\right)$, where w=μ+ε⋅σ, represents an element multiplication calculation. This can solve the problem of gradient non-transitivity. At the same time, in order to ensure that the value of the variational distribution parameter σ is greater than 0, the parameter σ is also resampled:

(8) $$\sigma =\log\left(1+e^{\rho }\right)$$

In this way, the distribution parameters of $q_{\phi }\left(w\right)$ become μ and ρ, which are different from the originally defined distribution parameters μ and σ. The uncertainty introduced by this sampling process can be regarded as global uncertainty. When performing Bayesian inference, it is necessary to sample all parameters globally. However, Diederik P. Kingma et al. [24] found that, if all parameters are independent Gaussian distributions, the results obtained through forward propagation will also be independent Gaussian distributions. This conclusion proves that we can directly calculate the forward-propagated values of the $q_{\phi }\left(w\right)$ distribution parameters for sampling, and then backpropagate them to the original distribution parameters for updating. The forward propagation values are calculated as follows:

(9) $$F_{\mu }=XW_{\mu }+B_{\mu }$$

(10) $$F_{\sigma }=XW_{\sigma }+B_{\sigma }$$

(11) $$F=F_{\mu }+\varepsilon \cdot F_{\sigma }$$

Among them, X represents the input data matrix of the model, $F_{\mu }$ and $F_{\sigma }$ correspond to the values of the $q_{\phi }\left(w\right)$ distribution parameters after the forward-propagation calculation, F represents the final return value of forward propagation, $W_{\mu }$ and $B_{\mu }$ represent the matrices corresponding to the mean parameter μ of the model weight and bias, and $W_{\sigma }$ and $B_{\sigma }$ represent the matrices corresponding to the standard deviation parameter $\log\left(1+e^{\rho }\right)$ of the model weight and bias after resampling. This method is called the local reparameterization technique (LRT) [24] because it does not require sampling of global parameters, and is often used in Bayes-by-backprop methods to reduce the gradient variance during training and prevent overfitting.

The objective function in Formula (4) and the approximation in Formula (5) are performed by the model on the entire dataset. However, in the actual implementation process, the small-batch gradient descent method is often used to divide the dataset into batches, so correspondingly, the complexity cost $KL\left[q_{\phi }\left(w\right)||\;p\left(w\right)\right]$ needs to be scaled. Assuming that the entire dataset is divided into M batches, the simplest scaling method is to average the complexity cost of each batch, and the corresponding objective function can be written as:

(12) $$F\left(D,\phi \right)=\frac{1}{M}KL\left[q_{\phi }\left(w\right)||\;p\left(w\right)\right]-E_{q_{\phi }\left(w\right)}\left[\log p\left(D|\;w\right)\right]$$

On this basis, Blundell et al. [12] proposed a new scaling method by modifying the proportion of the complexity cost in each batch, theoretically achieving the idea of focusing on fitting priors in the early stage of training and fitting data in the later stage. The corresponding objective function is written as follows:

(13) $$F\left(D,\phi \right)=\pi_{i}KL\left[q_{\phi }\left(w\right)||\;p\left(w\right)\right]-E_{q_{\phi }\left(w\right)}\left[\log p\left(D|\;w\right)\right]$$

with $\pi_{i}$ ∈ [0, 1] ^M^ in Formula (13), and $\sum_{i=1}^{M}\pi_{i}=1$, $\pi_{i}=\frac{2^{M-i}}{2^{M}-1}$.

This paper applies the local reparameterization technique mentioned above to the Bayes-by-backprop method for Bayesian inference, and scales the complexity cost calculation involved in it, as shown in (13). The sampling Adams optimizer minimizes the objective function to optimize the network parameters.

## 3. BCNN Framework and RUL Uncertainty Quantification

**Figure 1:**
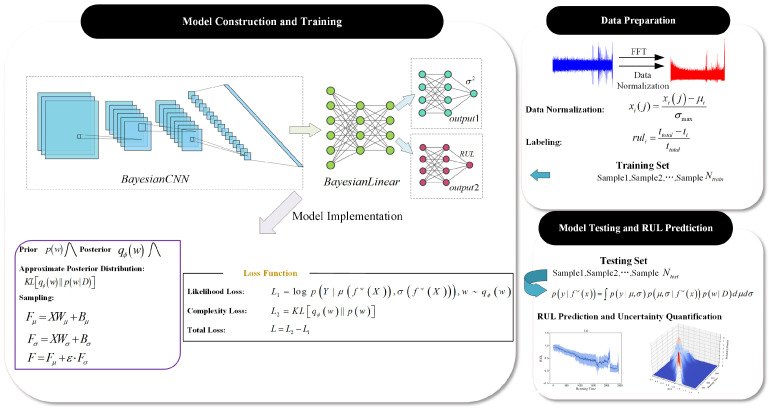
Method framework.

The Bayesian framework is applied to convolutional neural networks (CNNs) to achieve RUL uncertainty quantization, and the method proposed in this paper is shown in Figure 1. There were three main modifications: firstly, the model parameters in the convolutional and fully connected layers of the CNN were converted into random variables, and probability distributions were used to represent the epistemic uncertainty of the model. Secondly, a dual-output unit was set up behind the fully connected layer of the CNN to predict the point estimate of the RUL and the inherent variance in the data themselves. Finally, the negative logarithmic probability density function was used as part of the loss function to optimize the dual-output unit results, and the variance in the data themselves was obtained as aleatoric uncertainty. At the same time, the sum of the KL divergence was used as another part of the loss function to optimize the variational distribution parameters, approximately replacing the posterior distribution of parameters in order to obtain the predicted probability to quantify the epistemic uncertainty.

In the proposed BCNN model, the probability distributions in the convolutional and fully connected layers represent epistemic uncertainty, while the dual-output structure models heteroscedastic aleatoric uncertainty. When considering the life distribution parameterized by μ and σ, the likelihood function of the BCNN model in Formula (1) can be written as:

(14) $$p\left(y|\;f^{w}\left(x\right)\right)=\int p\left(y|\;\mu ,\sigma \right)p\left(\mu ,\sigma |\;f^{w}\left(x\right)\right)p\left(w|\;D\right)d\;\mu d\sigma$$

Among them, w represents the random variable of the model, corresponding to the posterior distribution of Formula (2), written as:

(15) $$p\left(w|\;D\right)=\frac{p\left(w\right)\int p\left(y|\;\mu ,\sigma \right)p\left(\mu ,\sigma |\;f^{w}\left(x\right)\right)d\;\mu d\sigma }{\int p\left(y|\;\mu ,\sigma \right)p\left(\mu ,\sigma |\;f^{w}\left(x\right)\right)p\left(w\right)d\;\mu d\sigma dw}$$

The model prediction probability corresponding to Formula (1) is:

(16) $$p\left(y^{*}|\;x^{*},D\right)=\int p\left(y^{*}|\;\mu ,\sigma \right)p\left(\mu ,\sigma |\;f^{w}\left(x^{*}\right)\right)p\left(w|\;D\right)d\;\mu d\sigma dw$$

From the previous description, it can be seen that the posterior distribution $p\left(w|\;D\right)$ usually cannot calculate an analytical solution, and variational inference theory needs to be used to approximate the posterior distribution $p\left(w|\;D\right)$ using the variational distribution $q\left(w\right)$. By substituting $q\left(w\right)$ into Formula (16), we can obtain:

(17) $$p\left(y^{*}|\;x^{*}\right)=\int p\left(y^{*}|\;\mu ,\sigma \right)p\left(\mu ,\sigma |\;f^{w}\left(x^{*}\right)\right)q\left(w\right)d\;\mu d\sigma dw$$

According to the Bayes-by-backprop method, minimizing the KL divergence of variational and posterior distributions is equivalent to maximizing the lower bound of evidence, and its optimization function can be expressed as

(18) $$\begin{array}{cc} L_{ELBO} & =\int q\left(w\right)\log\left(\int p\left(Y\;|\;\mu ,\sigma \right)p\left(\mu ,\sigma \;|\;f^{w}\left(X\right)\right)d\;\mu d\sigma \right)dw\\ & \;-KL\left[q\left(w\right)||\;p\left(w\right)\right] \end{array}$$

In the explanation of Part 2, it is known that the optimization function can obtain its unbiased estimate using the Monte Carlo sampling method, and the equation is:

(19) $$\begin{array}{cc} L_{MC} & =E_{q_{\phi }\left(w\right)}\left[\log\left(\int p\left(Y\;|\;\mu ,\sigma \right)p\left(\mu ,\sigma \;|\;f^{w}\left(X\right)\right)d\;\mu d\sigma \right)\right]\\ & \;-E_{q_{\phi }\left(w\right)}\left[\log q_{\phi }\left(w\right)\right]+E_{q_{\phi }\left(w\right)}\left[\log p\left(w\right)\right] \end{array}$$

In Formula (19), parameters μ and σ are generated by model weights, so $p\left(Y\;|\;\mu ,\sigma \right)p\left(\mu ,\sigma \;|\;f^{w}\left(X\right)\right)$ can be simplified to $p\left(Y\;|\;\mu \left(f^{w}\left(X\right)\right),\sigma \left(f^{w}\left(X\right)\right)\right)$. Substituting it into Formula (19) yields:

(20) $$\begin{array}{cc} L_{MC} & =E_{q_{\phi }\left(w\right)}\left[\log p\left(Y\;|\;\mu \left(f^{w}\left(X\right)\right),\sigma \left(f^{w}\left(X\right)\right)\right)\right]\\ & \;-E_{q_{\phi }\left(w\right)}\left[\log q_{\phi }\left(w\right)\right]+E_{q_{\phi }\left(w\right)}\left[\log p\left(w\right)\right] \end{array}$$

Among them, $w\;\sim \;q_{\phi }\left(w\right)$. By maximizing the lower bound of evidence as described above, the optimal variational distribution can be obtained to approximate the posterior distribution of the model. After the model training is completed, it is necessary to quantify the uncertainty of the RUL prediction results. According to the theory of variance accumulation [10], let the predicted variance $Var\left(y^{*}\;|x^{*},w\right)$ represent the sum of aleatoric uncertainty and epistemic uncertainty. Reconstructing the total predicted variance yields the following equation:

(21) $$\begin{array}{cc} Var\left(y^{*}|x^{*},D\right) & =E_{p\left(w\left|D\right.\right)}\left[{\left(y^{*}\right)}^{2}\right]-{\left[E_{p\left(w\left|D\right.\right)}\left(y^{*}\right)\right]}^{2}\\ & =E_{p\left(w\left|D\right.\right)}\left[E\left({\left(y^{*}\right)}^{2}|x^{*},w\right)\right]-{\left(E_{p\left(w\left|D\right.\right)}\left[E\left(y^{*}|x^{*},w\right)\right]\right)}^{2}\\ & =E_{p\left(w\left|D\right.\right)}\left[Var\left(y^{*}|x^{*},w\right)+{\left[E\left(y^{*}|x^{*},w\right)\right]}^{2}\right]-{\left(E_{p\left(w\left|D\right.\right)}\left[E\left(y^{*}|x^{*},w\right)\right]\right)}^{2}\\ & =E_{p\left(w\left|D\right.\right)}\left[{\left(E\left(y^{*}|x^{*},w\right)\right)}^{2}\right]-{\left(E_{p\left(w\left|D\right.\right)}\left[E\left(y^{*}|x^{*},w\right)\right]\right)}^{2}+E_{p\left(w\left|D\right.\right)}\left[Var\left(y^{*}|x^{*},w\right)\right]\\ & =Var_{p\left(w\left|D\right.\right)}\left[E\left(y^{*}|x^{*},w\right)\right]+E_{p\left(w\left|D\right.\right)}\left[Var\left(y^{*}|x^{*},w\right)\right] \end{array}$$

where $Var_{p\left(w\left|D\right.\right)}\left[\cdot \right]$ and $E_{p\left(w\left|D\right.\right)}\left[\cdot \right]$ denote the expectation and variance with respect to the posterior distribution of model parameters w, respectively.

The predicted total variance $Var\left(y^{*}|x^{*},D\right)$ in Formula (21) is decomposed into two parts, where $Var_{p\left(w\left|D\right.\right)}\left[E\left(y^{*}|x^{*},w\right)\right]$ represents the variance in the expected $E\left(y^{*}|x^{*},w\right)$, which can be used to represent the epistemic uncertainty, where the calculation of the expected value is influenced by the model parameters and data size. And $E_{p\left(w\left|D\right.\right)}\left[Var\left(y^{*}|x^{*},w\right)\right]$ eliminates the variability in the model parameters by calculating the expected value, which reflects the inherent uncertainty of the data itself, that is, the aleatoric uncertainty.

For a test sample $x^{*}$, the dual-output BCNN produces two outputs: the predictive mean $\mu \left(x^{*},w\right)$ and the predictive variance $\sigma^{2}\left(x^{*},w\right)$. Under the Gaussian likelihood assumption, the conditional predictive distribution of the RUL is given by $y^{*}|x^{*},w∼N\left(\mu \left(x^{*},w\right),\sigma^{2}\left(x^{*},w\right)\right)$. Accordingly, for a given model parameter sample w, the point prediction of the RUL is $\hat{y}\left(x^{*},w\right)=E\left[y^{*}|x^{*},w\right]=\mu \left(x^{*},w\right)$, and the corresponding conditional variance is $Var\left(y^{*}|x^{*},w\right)=\sigma^{2}\left(x^{*},w\right)$, which characterizes the aleatoric uncertainty caused by inherent noise in the data.

Since the model parameters w follow the posterior distribution $p\left(w|\;D\right)$, the overall predictive distribution is obtained by marginalizing over w: $p(y^{*}|\;x^{*},D)=\int p(y^{*}|\;x^{*},w)p(w|\;D)dw$. The predictive mean of the RUL is therefore given by ${\bar{y}}^{*}=E_{p(w|D)}[\mu (x^{*},w)]$, and the predictive variance can be decomposed according to the law of total variance as $Var(y^{*}|\;x^{*},D)=E_{p(w|\;D)}[\sigma^{2}(x^{*},w)]+Var_{p(w|\;D)}[\mu (x^{*},w)]$, where the first term represents the aleatoric uncertainty and the second term represents the epistemic uncertainty.

Based on the predictive mean and total predictive variance, the RUL prediction interval at confidence level (1 − α) is constructed as

(22) $$\left[{\bar{y}}^{*}-z_{1-\alpha /2}\sqrt{Var(y^{*}|\;x^{*},D)},{\bar{y}}^{*}+z_{1-\alpha /2}\sqrt{Var(y^{*}|\;x^{*},D)}\right]$$

where $z_{1-\alpha /2}$ is the standard normal quantile.

After training the BCNN model, its total prediction uncertainty can be obtained through Monte Carlo sampling, and its calculation formula is as follows:

(23) $$\begin{array}{l} Mean\left(y^{*}\right)=\frac{1}{M}\frac{1}{N}\sum_{m=1}^{M}\sum_{n=1}^{N}{\hat{y}}_{n}^{m}\\ Var\left(y^{*}\right)=\frac{1}{M}\frac{1}{N}\sum_{m=1}^{M}\sum_{n=1}^{N}{\left({\hat{y}}_{n}^{m}-Mean\left(y^{*}\right)\right)}^{2} \end{array}$$

In Formula (22), calculations are performed by multiple sampling of $w_{m}\sim \;q_{\phi }\left(w\right)$ and ${\hat{y}}_{n}^{m}\sim \;p\left(y\;|\;\mu_{m},\sigma_{m}\right)$, where the ranges of m and n are [1,M] and $\left[1,N\right]$, respectively. This form of double sampling is determined by the applied BCNN dual-output unit structure network. In Formula (21), the epistemic uncertainty decomposed from the total prediction uncertainty needs to be solved for the variance of the mean, while aleatoric uncertainty only needs to calculate the mean of the variance. Relatively speaking, for the convenience of calculation, the aleatoric uncertainty in the predicted results can be solved by calculating the average variance of each model weight, which is given by the formula:

(24) $$\begin{array}{l} Mean_{m}\left(y^{*}\right)=\frac{1}{N}\sum_{m=1}^{M}\sum_{n=1}^{N}{\hat{y}}_{n}^{m}\\ Var_{aleatoric}\left(y^{*}\right)=\frac{1}{M}\frac{1}{N}\sum_{m=1}^{M}\sum_{n=1}^{N}{\left({\hat{y}}_{n}^{m}-Mean_{m}\left(y^{*}\right)\right)}^{2} \end{array}$$

By substituting Formula (23) into Formula (21), the epistemic uncertainty can be calculated as follows:

(25) $$Var_{epistemic}\left(y^{*}\right)=Var\left(y^{*}\right)-Var_{aleatoric}\left(y^{*}\right)$$

The overall training, sampling, and variance decomposition procedure of the proposed dual-output BCNN is summarized in Algorithm 1.

**Algorithm 1.** Dual-output BCNN variance decomposition.Input: Training samples: $D_{train}={\left\{x_{i},y_{i}\right\}}_{i=1}^{N_{train}}$; testing samples: $D_{test}={\left\{x_{i}\right\}}_{i=1}^{N_{test}}$; Iterations: M and N; network parameters: ω;Output: Variational distribution $q_{\phi }\left(\omega \right)$; predicted mean μ; predicted variance σ;
For m from 1 to M, do

2.   Draw $\left(\mu_{m},\sigma_{m}\right)$ with model parameters $\omega_{m}∼q_{\phi }\left(\omega \right)$

3.   For *n* from 1 to *N*, do

4.      Draw sample ${\hat{y}}_{n}^{m}$ from $p\left(y\;|\;\mu_{m},\sigma_{m}\right)$

5.   End for

6.End for

7.Compute the $Mean\left(y^{*}\right)$ for ${\hat{y}}_{n}^{m}$ using (22)

8.Decompose the $Var\left(y^{*}\right)$ using (23) and (24)


## 4. Case Description

In order to verify the effectiveness and superiority of the Bayesian convolutional neural network (BCNN) framework proposed in this paper for measuring the uncertainty in RUL predictions, this section uses the IEEE PHM 2012 dataset for experimental verification. The algorithm in this paper was trained on the Windows 10 system and an NVIDIA GeForce RTX 4060 GPU.

### 4.1. Overview of the Dataset

**Figure 2:**
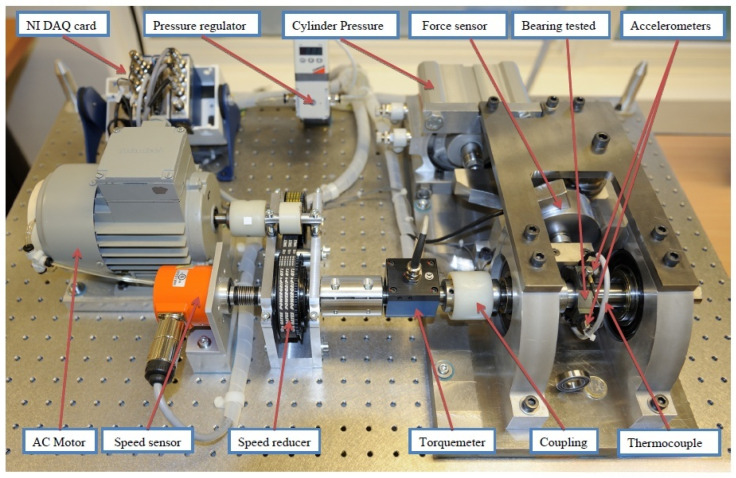
The PRONOSTIA testbed of rolling element bearings.

The IEEE PHM 2012 dataset was provided by the FEMTO-ST Institute and experiments were conducted on the experimental platform (PRONOSTIA). This experimental platform can meet the requirements of applying different working conditions to the test bearings and collect real-time bearing health monitoring data, as shown in Figure 2.

The PRNONSTIA platform consists of three parts: the rotating part, the load part, and the testing part. The rotating part is powered by a 250 W AC motor to apply torque to the shaft, ensuring that the second shaft reaches a speed of 2000 rpm. The load part applies a radial dynamic load of 4000 N to the tested bearing through a pneumatic jack, accelerating the degradation process of the bearing. The testing section is used to collect complete bearing degradation data, including collecting bearing vibration data and temperature data. For this purpose, vibration sensors are placed radially and axially on the outer ring of the bearing to measure data in both directions, with a sampling frequency of 25.6 kHz. Meanwhile, a resistance temperature detector is placed in the hole near the outer bearing ring to collect temperature data, with a sampling frequency of 0.1 Hz.

When conducting an RUL prediction analysis on bearings, the main data used are the vibration data of the bearing degradation process. This dataset contains horizontal and vertical vibration signals under three working conditions. The sensor collects signals every 10 s with a recording length of 0.1 s, and each collected vibration signal contains 2560 data points. During the entire bearing data collection process, for safety reasons, testing is stopped when the amplitude of the vibration signal exceeds 20 g. The statistics of the three operating conditions and the tested bearings are shown in Table 1.

### 4.2. Data Processing and Evaluation Indicators

**Figure 3:**
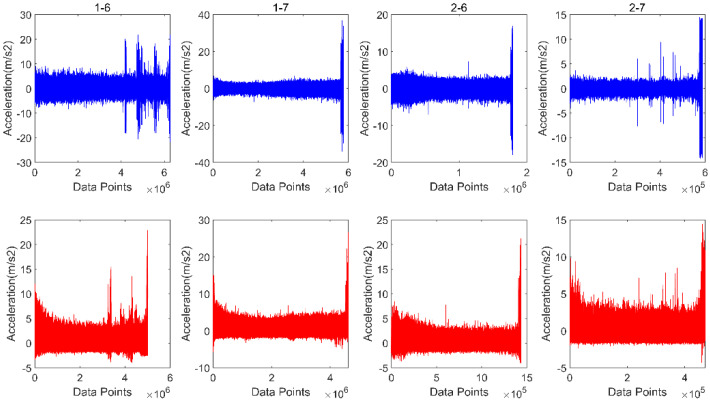
Time-domain and frequency-domain display of vibration data.

To augment the training dataset, bearings 1-6, 1-7, 2-6, and 2-7 from operating conditions 1 and 2 were allocated to the test set, while the remaining bearings constituted the training set. Due to the unclear degradation features contained in the original vibration signal, in order to improve the training effect, it is necessary to perform a spectral analysis and z-score standardization on the dataset, and use the standardized data as the input for neural network training and testing. A comparison between the original vibration signal of the data and the standardized signal of the spectral analysis is shown in Figure 3.

When assigning RUL labels to the dataset, linearly decreasing the RUL over time, and considering the differences in the service life of different bearings, setting the RUL range to 0–1 can achieve better training results.

To verify the effectiveness of the proposed method, the accuracy [25] and root mean square error (RMSE) were used as evaluation metrics for the RUL point estimation accuracy. Among them, accuracy represents the accuracy of the RUL prediction, and the larger the accuracy value, the higher the prediction accuracy; the RMSE reflects the average deviation between the predicted values and true values, with lower values indicating a better prediction performance. The calculation formulas are as follows:

(26) $$Accuracy=\frac{1}{N}\sum_{i=1}^{N}e^{\frac{-|m_{i}|}{RUL\_True}}$$

(27) $$RMSE=\sqrt{\frac{1}{N}\sum_{i=1}^{N}m_{i}^{2}}$$

Among them, *m_i_* is the prediction error value at the i-th time point, calculated as *m_i_* = predicted remaining life − true remaining life; N is the number of data collection points.

In addition, in order to evaluate the performance of model uncertainty quantification, two indicators were used: the prediction interval coverage probability, PCIP, and the average prediction interval width, MPIW. Among them, PCIP represents the probability that the predicted interval contains the target value, and the larger its value, the higher the probability that the predicted target value is in the predicted interval. For uncertainty quantification goals, it is required that the PCIP value be large while the MPIW value be as small as possible, indicating a higher prediction accuracy. The calculation formula is as follows:

(28) $$PCIP=\frac{1}{N}\sum_{i=1}^{n}rul_{i}$$

(29) $$MPIW=\frac{1}{n}\sum_{i=1}^{n}\left(H_{i}-L_{i}\right)$$

Among them, if, and only if, $L_{i}\leq RUL_{i}\leq H_{i}$, $rul_{i}=1$; in other cases, $rul_{i}=0$. L_i_ and H_i_ represent the lower and upper limits of the prediction interval, respectively.

### 4.3. Experimental Analysis

The BCNN model transforms the training parameters in traditional CNNs into random variables without using the dropout method for uncertainty substitution. The network parameter graph is shown in Table 2.

**Figure 4:**
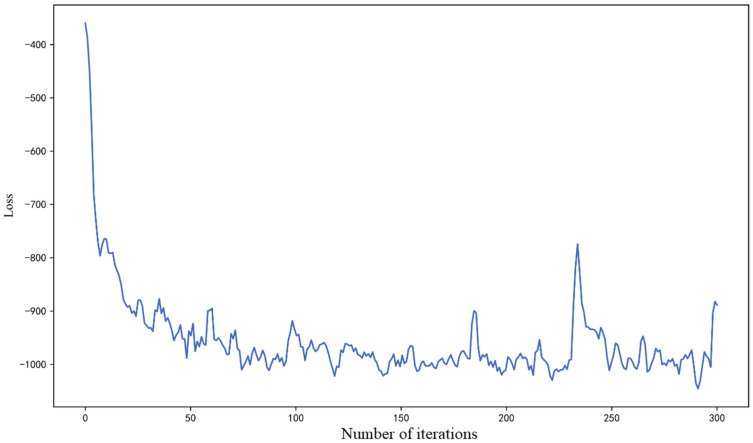
Training loss diagram of network model.

The BConv1d and BL layers used in BCNNs are Bayesian convolutional layers and Bayesian linear layers, respectively. They are the Conv1d and linear layers rewritten using Bayesian principles. The hyperparameters of the neural network were tuned using the Bayesian optimization algorithm Hyperopt, and the resulting hyperparameter values are shown in Table 3. The model training loss curve obtained using the following network configuration is shown in Figure 4. The negative loss values observed in Figure 4 were mainly due to the negative log-likelihood (NLL) term. For continuous distributions such as the Gaussian likelihood, the probability density may exceed 1, leading to positive log-likelihood values, and thus, negative NLL values. Although the KL divergence term is non-negative, the NLL term may dominate during training, resulting in an overall negative loss. This behavior is expected and does not indicate numerical instability. It was observed that the loss curve gradually approached a stable value at epoch 75, indicating the rationality of the training.

**Figure 5:**
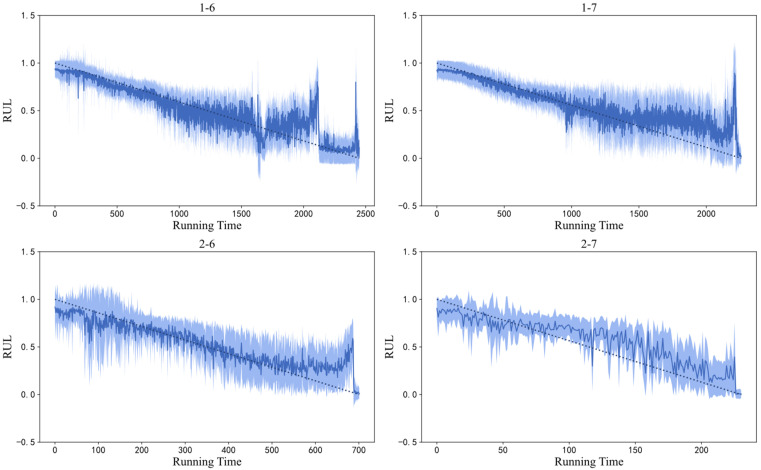
Unsmoothed image of the test set.

**Figure 6:**
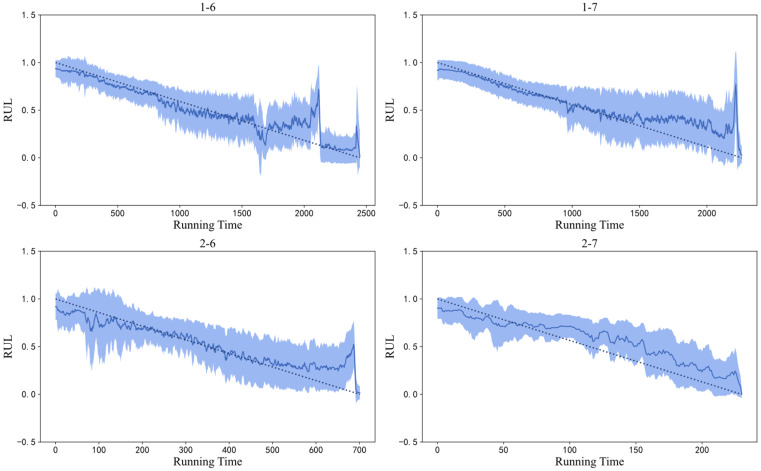
Smooth processing of test set images.

Using the training set described in Section 4.2 to train the proposed BCNN dual-output network, the original prediction performance of the test set is shown in Figure 5. In Figure 5, the original signal curve is close to the target value, indicating a relatively ideal prediction effect. However, there is a lot of signal stacking, and the overall trend observation is not obvious. In order to better observe and analyze the prediction performance, it is necessary to smooth the predicted data. It should be emphasized that sliding-window smoothing is used solely for visualization and does not influence the quantitative evaluation. Considering the significant differences in the number of data points in different test sets, the sliding window size for test sets 1-6 and 1-7 was selected as 10, while the sliding window size for test sets 2-6 and 2-7 was selected as 5. The resulting image is shown in Figure 6.

**Figure 7:**
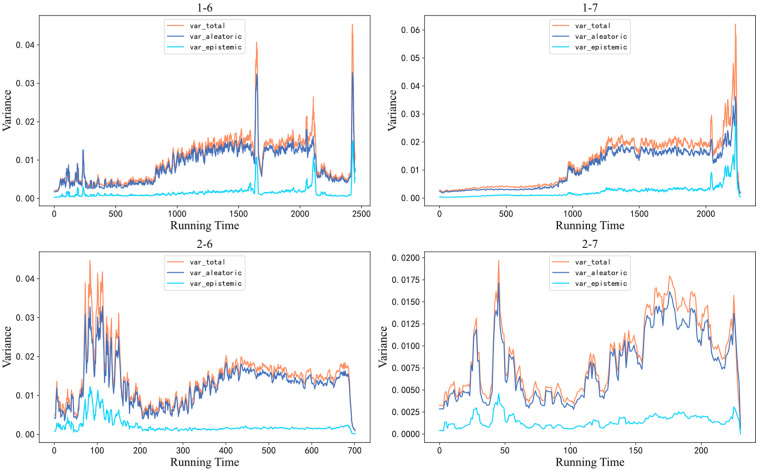
Test bearing uncertainty measurement diagram.

Based on Figure 5 and Figure 6, it can be seen that the BCNN dual-output network had a good performance in measuring the uncertainty in bearing predictions. For testing bearings 1-6 and 1-6, in the early stage of the RUL, the degradation information contained in their vibration data was not obvious, and the data distribution was roughly the same as that of the training set, so the data itself had a low uncertainty. In the mid-term stage of the RUL, considering that individual bearings have different assembly and stress conditions, the noise signals generated during the rotation process varied from individual to individual. Compared with the training set, the uncertainty of the data itself increased, resulting in significant uncertainty in the predicted curve at this stage. As the bearing approached the degradation stage, due to the rapid degradation of the bearing, the degradation information that was contained increased. This situation also existed for all bearings in the training set, so their data distributions represented more degradation information, resulting in a lower uncertainty of the test data itself and a lower uncertainty of the prediction curve. This observation is important for equipment health monitoring, as an accurate prediction in the later stage is crucial for monitoring. The uncertainty mentioned in the above analysis mainly refers to aleatoric uncertainty. For epistemic uncertainty, due to the fact that the amount of data input in the training set is much larger than that in the test set, and the amount of data in the training set remains unchanged, the proportion of epistemic uncertainty is relatively small compared to aleatoric uncertainty. The presented image effect is shown in Figure 7.

For testing bearings 2-6 and 2-7, there was significant uncertainty in the early stages of the RUL, possibly due to the individual bearing being more prone to minor failures during the early break in the stage, including obvious degradation information, resulting in a shorter overall lifespan. This phenomenon was caused by the unique assembly or stress conditions of the individual bearing, and it belongs to a special case. However, in the mid- to late stages, testing bearings 2-6 and 2-7 met the conditions analyzed above. As the bearings approached the degradation stage, their uncertainty decreased, which played a good role in bearing monitoring.

**Figure 8:**
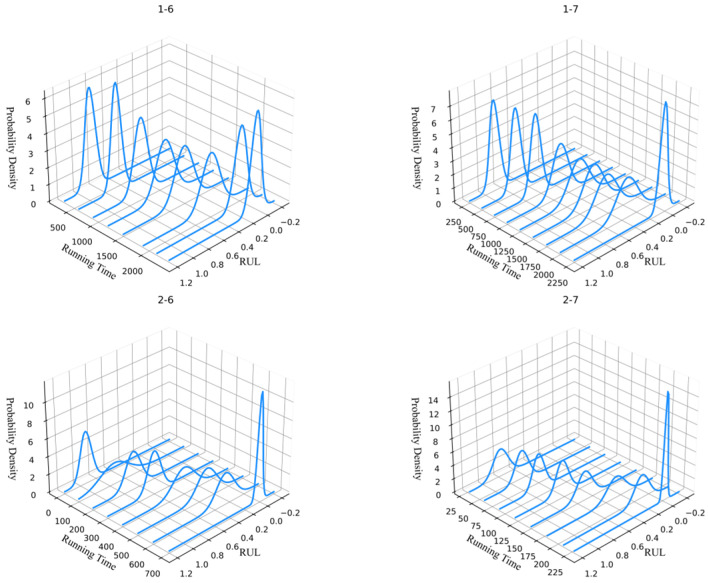
Test bearing PDF diagram.

As shown in Figure 5 and Figure 8, it can be seen that the RUL prediction performance of the method in this chapter was relatively accurate, and the confidence interval of the tested bearings included the vast majority of the target values. All four tested bearings had good predictive values near the degradation stage, almost 100% predicting the degradation failure point of the bearings. There was a certain gap between the predicted and ideal values of bearings 2-7 in the mid-term RUL stage, but their overall degradation trend remained consistent with the target value and the method still had a good prediction performance. In summary, the method proposed in this paper can effectively achieve RLU predictions and an uncertainty analysis of bearings.

### 4.4. Contrast Test

To evaluate the effectiveness of the proposed method, comparative experiments were conducted with several representative models for RUL predictions and uncertainty quantification, including a dual-output CNN, a single-output BCNN (Bayes-by-backprop), an MC dropout BCNN, and additional state-of-the-art architectures such as CNN-LSTM and transformer-BiLSTM variants. All the models were trained under the same experimental conditions to ensure a fair comparison. For clarity, the comparison focused on the test bearing 1-6, and the evaluation metrics defined in Section 4.2 were adopted. The quantitative comparison results are summarized in Table 4, including the prediction performance (RMSE and accuracy), uncertainty evaluation metrics (PCIP and MPIW), and computational complexity indicators (FLOPs and parameter size).

From the results, it can be observed that the proposed dual-output BCNN achieved the best overall performance in uncertainty quantification. Specifically, it attained the highest PCIP value of 0.9628, and significantly outperformed all other methods. This indicates that the prediction intervals generated by the proposed model can effectively cover the true RUL values, providing more reliable decision support for practical applications. Although the MPIW of the proposed method was relatively larger, this is a reasonable trade-off, as a higher coverage probability is more critical in safety-related engineering scenarios.

Compared with the dual-output CNN, which introduces a dual-output structure to capture heteroscedastic uncertainty, the proposed method further incorporates Bayesian inference to model the epistemic uncertainty. As a result, it significantly improves both the prediction accuracy and interval reliability. The relatively low PCIP value of the dual-output CNN indicates that modeling only the aleatoric uncertainty is insufficient for reliable RUL predictions.

For the single-output BCNN and MC dropout BCNN, both methods attempt to quantify the epistemic uncertainty through Bayesian approximation. However, due to the absence of a dual-output structure, they fail to explicitly model the aleatoric uncertainty. Consequently, although they achieve relatively low MPIW values, their PCIP values remain limited, indicating that their prediction intervals cannot adequately capture the true RUL values. This further demonstrates the necessity of jointly modeling both types of uncertainty.

In addition, to enhance the comprehensiveness of the comparison, advanced sequence modeling architectures, including CNN-LSTMs and Transformer-BiLSTMs, were incorporated. These models leverage temporal feature extraction capabilities and show a competitive performance in their point prediction accuracy. For example, the single-output transformer-BiLSTM achieved a relatively low RMSE compared with conventional CNN-based models.

However, these models either lack uncertainty quantification mechanisms or exhibit an unsatisfactory uncertainty performance, as reflected by missing or relatively low PCIP values. Even for their dual-output variants, the PCIP values remained significantly lower than that of the proposed method, indicating a limited reliability in interval prediction. This suggests that simply increasing the model complexity or introducing temporal modeling does not guarantee effective uncertainty quantification.

From the perspective of computational complexity, the proposed dual-output BCNN introduces additional overhead due to Bayesian inference and variational parameterization. As shown in Table 4, its FLOPs and parameter size are approximately twice those of the standard CNN-based models. However, compared with more complex architectures such as CNN-LSTMs and Transformer-BiLSTMs, the proposed method maintains a moderate computational cost. More importantly, this increase in the computational complexity leads to substantial improvements in the uncertainty quantification performance, particularly in PCIP. Therefore, the proposed method achieves a favorable balance between computational efficiency and predictive reliability.

Overall, the experimental results demonstrate that the proposed dual-output BCNN not only achieves a competitive performance in point predictions compared with state-of-the-art models, but it also significantly outperforms them in uncertainty quantification. This makes it more suitable for practical PHM applications where both the prediction accuracy and reliability are essential.

## 5. Summary

This article proposes a dual-output unit Bayesian CNN that uses the Bayes-by-backprop method for variational inference for a Bayesian analysis. Meanwhile, the traditional single-output unit is replaced with a dual-output unit to simulate the distribution of the output data. Compared to other uncertainty quantification methods, the method proposed in this paper can capture and quantify epistemic uncertainty caused by data scarcity or model parameters, while utilizing the advantages of dual-output units to quantify any heteroscedastic uncertainty.

To verify the effectiveness of the proposed method, this paper conducted experimental validation using the PHM2012 dataset. The experimental results show that the method proposed in this paper can effectively complete the prediction and uncertainty quantification process of the RUL. In addition, by quantifying two types of uncertainties, the change process of the RUL prediction confidence interval was analyzed, providing a more valuable reference for equipment monitoring and maintenance. By comparing with other traditional uncertainty quantification methods, it was found that uncertainty quantification using our method almost completely includes the target value in the confidence interval, and has an average improvement of 28% on the PCIP index. It can be seen that the method proposed in this paper is superior to traditional uncertainty quantification methods in terms of uncertainty quantification. In addition, the ability to quantify two types of uncertainty provides more interpretable recommendations for monitoring and maintenance.

## Figures and Tables

**Table 1:** The statistics of the operating conditions and the tested bearings.

Working Conditions	Load: 4000 Speed: 1800 rpm	Load: 4200 Speed: 1650 rpm	Load: 5000 Speed: 1500 rpm
Learning set	Bearing 1-1	Bearing 2-1	Bearing 3-1
	Bearing 1-2	Bearing 2-2	Bearing 3-2
Test set	Bearing 1-3	Bearing 2-3	Bearing 3-3
	Bearing 1-4	Bearing 2-4	
	Bearing 1-5	Bearing 2-5	
	Bearing 1-6	Bearing 2-6	
	Bearing 1-7	Bearing 2-7	

**Table 2:** Network architecture diagram.

Block	Layer	Filter	Feature Size
1	Input		1 × 2048
	BConv1d ReLU()	In = 3, out = 8, k = 8	3 × 2041
	MaxPool1d	Size = 3	3 × 680
2	Same as Block 1	In = 3, out = 6	6 × 224
3	Same as Block 1	In = 6, out = 8	8 × 72
4	Same as Block 1	In = 8, out = 16	16 × 21
5	Flatten	/	336
6	BL	336 × 200	200
7	BL	200 × 200	200
8	BL Exp()	200 × 1	1
9	BL Sigmoid()	200 × 1	1

**Table 3:** The hyperparameters in the dataset.

Hyperparameters	IEEE PHM 2012
Train epoch	75
Prior distribution	N (0, 0.1)
Learning rate	0.01
Batch size	512

**Table 4:** Comparison results of model complexity and performance indicators.

Method	FLOPs	Params	RMSE	ACCURACY	PCIP	MPIW
Dual-output CNN	5.135 M	109.677 K	0.1253	0.7255	0.7304	0.2701
Single-output BCNN	10.175 M	218.8 K	0.0981	0.8009	0.7484	0.1977
MC dropout BCNN	5.135 M	109.677 K	0.0901	0.7987	0.7496	0.1686
Dual-output BCNN (proposed method)	10.176 M	219.2 K	0.0894	0.8061	0.9628	0.3740
Single-output CNN-LSTM	30.868 M	68.801 K	0.1297	0.7560	*	*
Dual-output CNN-LSTM	40.304 M	101.890 K	0.1180	0.7667	0.6299	0.1836
Single-output transformer-BiLSTM	46.277 M	67.169 K	0.1109	0.7675	*	*
Dual-output transformer-BiLSTM	46.310 M	67.234 K	0.1283	0.7557	0.7259	0.2026

* Indicates that this indicator is not applicable to the model.

## Data Availability

Data available on request from the authors.
